# Reduction of Asthmatic Parameters by Sea Hare Hydrolysates in a Mouse Model of Allergic Asthma

**DOI:** 10.3390/nu9070699

**Published:** 2017-07-05

**Authors:** Ji Hyeon Ryu, Chengliang Xie, Eun-Jin Kim, Si-Hyang Park, Yeung Joon Choi, Sang Soo Kang, Min-Kyoung Shin, Dawon Kang

**Affiliations:** 1 Department of Convergence Medical Science, Gyeongsang National University, Jinju 52727, Korea; wlgus9217@naver.com; 2 Department of Physiology and Institute of Health Sciences, College of Medicine, Gyeongsang National University, Jinju 52727, Korea; eunjin1981@hanmail.net; 3 Department of Anatomy and Institute of Health Sciences, College of Medicine, Gyeongsang National University, Jinju 52727, Korea; xie830734@naver.com (C.X.); kangss@gnu.ac.kr (S.S.K.); 4 Sunmarin Biotech, Tongyeong 53064, Korea; hyangi51@hanmail.net; 5 Department of Seafood Science and Technology and Institute of Marine Industry, Gyeongsang National University, Tongyeong 53064, Korea; yjchoi@gnu.ac.kr; 6 Department of Microbiology and Institute of Health Sciences, College of Medicine, Gyeongsang National University, Jinju 52727, Korea

**Keywords:** airway smooth muscle cell, asthma, cytokines, sea hare hydrolysates

## Abstract

Sea hare has a variety of biological activities. However, little is known regarding the anti-asthmatic effects of sea hare. This study was performed to identify the effect of sea hare hydrolysates (SHH) on an ovalbumin (OVA)-induced allergic asthma model. The experimental asthma model was sensitized and challenged with OVA. We found that a high-dose of SHH (HSHH) significantly inhibited OVA-induced airway inflammation and mucus production around the airway in lung sections, while low- and medium-dose SHH showed an insignificant effect. In addition, HSHH highly reduced OVA-induced production of interleukin-4, -5, -13, leukotriene D4, E4, and histamine in bronchoalveolar lavage fluid. HSHH decreased the histamine-induced increase in the intracellular Ca^2+^ level and contractions in asthmatic smooth muscle cells. Furthermore, HSHH did not affect the weights of the spleen nor thymus, whereas dexamethasone (DEX), a steroidal anti-inflammatory drug, reduced them. Taken together, these results showed that HSHH reduced asthmatic parameters in a mouse model of allergic asthma, and suggest that SHH could be used as a potential therapeutic agent for asthma.

## 1. Introduction

Sea hare, *Aplysia kurodai (A*. *kurodai)*, is a type of shell-less and slow-moving marine mollusk, which is commonly found in rocky seashores and is well-known in Asia as an edible marine organism [1,2]. Sea hare has a large number of bioactive molecules, and secretes the molecules as a defense mechanism to compensate for the absence of a shell as physical protection [3]. The bioactive molecules have shown a variety of functions, such as antitumor, antioxidant, antimicrobial, antibacterial, and immunostimulatory activities [4,5,6,7].

Asthma is an obstructive respiratory disorder that is characterized by an increased infiltration of leukocytes, specifically eosinophils (Eos’s), airway inflammation, bronchoconstriction, mucus hypersecretion, and airway hyperresponsiveness [8,9]. The inflammatory response of asthma is driven by an imbalance between T helper cell type 1 (Th1) and T helper cell type 2 (Th2) immune responses, with a bias towards an increase in the Th2 immune response [8]. Activated Th2 cells within the lungs produce various cytokines, such as IL-4, IL-5, and IL-13, which promote immunoglobulin E (IgE) production; induce the differentiation, maturation, and migration of Eos’s; and enhance airway hyperresponsiveness [8,10,11]. In addition, Th2 cytokine-activated mast cells release many bronchoconstrictor mediators, such as histamine, prostaglandin D2, and cysteinyl-leukotrienes [12,13,14]. An inhibition of Th2 cytokines and bronchoconstrictor mediators may suppress airway inflammation and ameliorate the symptoms of asthma. 

According to the global asthma report in 2014, the number of people suffering from asthma has been estimated at 334 million, and its prevalence is rapidly increasing. Asthma causes dynamic heterogeneous clinical syndromes with different patterns, including recurrent coughing, breathlessness, wheezing, damage of lung function, sleep disturbances, limitations in activities of daily living, and the use of rescue medicine [15]. These features may reduce the quality of life, and could cause asthma attacks and death in people with asthma. A wide range of pharmacological therapies has been developed for medications to treat asthma. The current asthma treatment employs a combination of medicines, including bronchodilators, leukotriene modifiers, mast cell stabilizers, and inhaled glucocorticosteroids (ICSs) [15,16]. However, many patients have concerns regarding the side effects of conventional medication for asthma. In particular, in the case of ICS treatment as one of the common treatments, the long-term treatment with high-doses of ICSs has been proposed to cause systemic side effects [15,16]. Therefore, seeking alternatives for the long-term, safe and effective treatment for asthma is strongly needed. Mouse models of allergic responses to inhaled ovalbumin (OVA) have been widely used for seeking potential therapeutic drugs that reduce asthmatic parameters and features [17,18].

Marine organisms have been given more attention in the discovery of drug leads and in the development of functional foods due to the bioactive compounds derived from marine organisms, which show a wide range of biological activities, and play an important role in the treatment of human diseases [19]. However, little is known regarding the effect of sea hare (*A*. *kurodai*) hydrolysates (SHH) on asthma. Hydrolysates could have more bioactive molecules, which are released from the native protein by the proteolytic action of enzymes, compared to extracts [20]. This study was performed to investigate the effects of SHH on an OVA-induced allergic asthma model. 

## 2. Materials and Methods 

### 2.1. Ethical Approval

All experiments were performed with the approval of the Ethics Committee of Gyeongsang National University (GNU-151208-M0068).

### 2.2. Chemicals

All of the chemicals used in this study were purchased from Sigma (St. Louis, MO, USA), unless otherwise specified. Stock solutions of OVA (50 mg/mL) and dexamethasone (DEX; 5 mg/mL) were prepared in distilled water and dimethyl sulfoxide (DMSO), respectively. A solution containing an equivalent DMSO concentration was used as a control when DMSO was used as a solvent.

### 2.3. Preparation of Sea Hare Hydrolysates (SHH)

The sea hare was washed and blanched for 10 min without water after removing the guts. The blanched sea hare was minced using a meat grinder (M-12S, Hankook fujee Industries Co., Ltd., Suwon, Korea). Two volumes of water were added to the minced sea hare, and flavourzyme (2%) was then added. Flavourzyme was directly added to the minced sea hare to utilize physiologically active substances released from both mucilage and proteins, which are abundant in sea hare, and to reduce the manufacturing processes for functional molecules. The minced sea hare in the enzyme solution was adjusted to pH 6.0 with citric acid powder, incubated at 60 °C for 15 h, boiled at 100 °C for 10 min to inactivate the enzyme reaction, and then filtered with a 200 mesh screen sieve. The filtered solution was concentrated to Brix 50 using a rotary vacuum evaporator (WCR-P6, Daihan Scientific Co., Ltd., Wonju, Korea). Three volumes of ethanol were added to the concentrated hydrolysates. The hydrolysate solution was stirred and left to stand for 12 h until precipitation. The precipitate was freeze-dried, ground into powder, and stored at −70 °C until further analysis. At the time of the experiment, the dried materials were dissolved in distilled water at the indicated concentrations.

### 2.4. Experimental Animals

Female C57BL/6 mice (6 weeks old) were purchased from Koatech Co. (Animal Breeding Center, Pyongtaec, Korea). The mice were maintained in an animal facility under standard laboratory conditions for 1 week prior to the experiments. The mice were housed under a 12 h light/dark cycle in a specific pathogen-free area with food and water freely available. Body weights of the mice were measured weekly for 3 weeks and on the day of operation. Animal experiments were performed according to the guidelines of the Gyeongsang National University Animal Care and Use Committee (GNU-151208-M0068).

### 2.5. Experimental Asthma Models and Intervention

**Figure 1:**
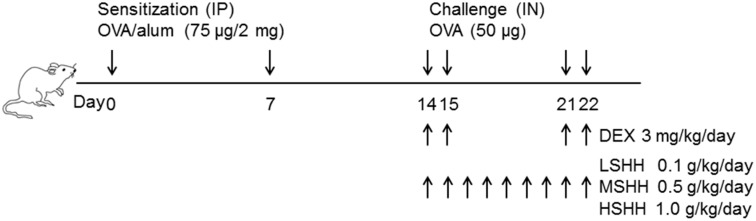
Modeling allergic asthma in mice. Mice were sensitized on days 0 and 7 with an intraperitoneal (IP) injection of 75 μg of ovalbumin (OVA) emulsified with 2 mg of aluminum hydroxide (alum) in 100 μL of phosphate-buffered saline (PBS). After the second sensitization, mice were challenged intranasally (IN) with 50 μg of OVA on days 14, 15, 21, and 22. Control mice were sensitized and challenged with PBS at the time of OVA injection. Control, OVA, and dexamethasone (DEX) groups received distilled water at the time of sea hare hydrolysates (SHH) administration. DEX and SHH were received orally 1 h before each OVA challenge (10:00–11:00 a.m.). The low-, medium-, and high-dose (LSHH, MSHH, and HSHH) groups orally received 0.1 g/kg, 0.5 g/kg, and 1.0 g/kg of SHH daily from day 14 to day 22. Animal experiments were performed in triplicate.

All mice were randomly divided into six groups (each group with *n* = 10) as follows: control, OVA, OVA + DEX (3 mg/kg/day), OVA + low-dose of SHH (LSHH; 0.1 g/kg/day), OVA + medium-dose of SHH (MSHH; 0.5 g/kg/day), and OVA + high-dose of SHH (HSHH; 1.0 g/kg/day). The actual dose for each treatment was calculated from the weights of the mice at the beginning of the experiments. Sensitization, challenge, and treatment protocols for the different groups in this study are summarized in Figure 1. This schedule is a classical and popular protocol for the studying of allergic responses in the airways [17,18,21]. Briefly, mice for the asthma models were sensitized on days 0 and 7 by an intraperitoneal (IP) injection of 75 μg of OVA emulsified with 2 mg of aluminum hydroxide (alum; InvivoGen, San Diego, CA, USA) in 100 μL of phosphate-buffered saline (PBS; pH 7.4). After the second sensitization, the mice were slightly anesthetized with avertin tribromoethanol (250 mg/kg), and intranasally (IN) challenged with 50 μg of OVA in PBS on days 14, 15, 21, and 22. The control mice were sensitized and challenged with PBS at the time of OVA injection. During the OVA challenge, different concentrations of SHH were orally administrated daily from day 14 to day 22. DEX, a medicine using for asthma treatment, was administered orally 1 h before each OVA challenge. The control mice were orally administered with distilled water daily from day 14 to day 22 at the time of SHH administration. All treated mice were sacrificed 24 h after the last challenge. Their spleens and thymi were isolated and weighed on the day of the operation. 

### 2.6. Collection of Bronchoalveolar Lavage Fluid (BALF) and Differential Cell Count

A tracheotomy was performed in mice anesthetized with avertin (250 mg/kg) 24 h after the last challenge. To obtain bronchoalveolar lavage fluid (BALF), 1 mL of PBS was infused into the lung twice and withdrawn each time via tracheal cannulation. The collected BALF was used for total and differential cell counts and cytokine analyses. Total cells counts in BALF were determined using a hemocytometer, and the BALF was centrifuged for 10 min at 2451× *g* at 4 °C. The supernatant was frozen at −20 °C for further cytokine analysis. Cells in BALF were attached on a slide by centrifugation (Cyto-tek, Sakura-Finetek Europe, Zoeterwoude, The Netherlands). The cell smear on a slide was stained with Wright-Giemsa solution, and 300 cells on the slide were quantified and classified into macrophages, lymphocytes, neutrophils (Neu’s), and Eos’s. The differential cell counting was performed based on standard morphological criteria [22,23].

### 2.7. Hematoxylin and Eosin (H&E) and Periodic Acid–Schiff (PAS) Stains

The mice were perfused with a fixative solution containing 4% paraformaldehyde in 0.1 M PBS. The left lungs were isolated, placed into a tube containing the same fixative solution, and incubated overnight at 4 °C. After washing three times, the lungs were processed in preparation for paraffin embedding and cut into 5 μm-thick sections. Lung inflammation was analyzed through Hematoxylin and Eosin (H&E) staining. Paraffin sections were air-dried on gelatin-coated slides, deparaffinized, and washed with tap water for 5 min. The deparaffinized sections were immersed in hematoxylin for 5 min, and checked for complete staining in tap water. Eosin staining was performed for 2 min. Mucus production was analyzed by periodic acid–Schiff (PAS) staining. Deparaffinized sections were immersed in a periodic acid solution for 5 min and then in Schiff’s reagent for 15 min. Hematoxylin was used for the counterstain in PAS staining. The stained sections were dehydrated through a graded series of alcohols (70–100% ethanol, 3 min each), cleared in xylene, and mounted with coverslips. The sections were imaged using a BX-51 microscope (Olympus, Tokyo, Japan), and five sections from each sample were evaluated. 

Infiltration of inflammatory cells in the peribronchial region was determined as described by Myou et al. [24]. The five-point scoring system was used: normal (0), a few cells (1), a ring of cells 1 cell layer-deep (2), a ring of cells 2–4 cell layers-deep (3), and a ring of cells >4 cell layers-deep (4). PAS-positive mucus-producing cells in the airway epithelium were quantified as described by Ford et al. [25]. The five-point scoring system was used: <5% PAS-positive cells (0); 5–25% (1), 25–50% (2), 50–75% (3), >75% (4). 

### 2.8. Measurement of Total and OVA-Specific Immunoglobulin E (IgE) in Serum

The levels of total and OVA-specific IgE in the serum were measured using the Mouse IgE ELISA Kit (KOMA Bio-tech., Seoul, Korea) and Anti-Ovalbumin IgE ELISA Kit (Cayman Chemical, Ann Arbor, MI, USA), respectively, according to the manufacturer’s instructions. Briefly, for measurements of both IgE, a 96-well plate pre-coated with mouse IgE antibody or mouse OVA-specific IgE antibody was washed five times with wash buffer, and 100 μL of serum obtained from each mouse was added to the 96-well plate. The plate was covered with the plate sealer, incubated for 1 h at room temperature on an orbital shaker, and washed five times with wash buffer.

After washing, for the measurement of the total IgE, 100 μL of detection antibody was added to the plate. The plate was incubated for 1 h at room temperature, and washed five times. Next, 100 μL of color development reagent was added to the plate. The plate was incubated for 15 min at room temperature, and the reaction was quenched by the addition of 100 μL of stop solution. For the measurement of the OVA-specific IgE, 100 μL of OVA-biotin conjugate working solution was added to the 96-well plate. The plate was incubated for 1 h at room temperature on an orbital shaker, and washed four times. Then, 100 μL of the streptavidin-linked horseradish peroxidase (SA-HRP) working solution was added to the plate. The plated was covered with the plate sealer, incubated for 30 min at room temperature on an orbital shaker, and washed four times. Next, 100 μL of tetramethylbenzidine (TMB) substrate solution was added to the plate, and the plate was incubated for 30 min at room temperature on an orbital shaker. The reaction was quenched by the addition of 100 μL of HRP stop solution. 

The absorbance of the total and OVA-specific IgE in the plates was read at 450 nm with a microplate reader (Molecular Devices, Sunnyvale, CA, USA).

### 2.9. Immunoassay for T Helper cell Type 2 (Th2) Cytokines, Leukotrienes, and Histamine in BALF

Concentrations of Th2 cytokines (IL-4, IL-5, and IL-13; R&D System, Minneapolis, MN, USA), leukotrienes (LTD4 and LTE4; MyBioSource, San Diego, CA, USA), and histamine (Enzo Life Sciences, Ann Arbor, MI, USA) in BALF were quantified using ELISA kits according to the manufacturer’s instructions. Briefly, for Th2 cytokines, 50 μL of BALF was added to the 96-well plates, which were pre-coated with anti-IL-4, -IL-5, or -IL-13 antibody. The plates were covered with an adhesive strip, incubated for 2 h at room temperature, and washed three times with wash buffer. Next, 100 μL of mouse IL-4, IL-5, or IL-13 conjugate was added, incubated for 2 h at room temperature, and washed three times. The reaction was quenched by the addition of 100 μL of stop solution.

For the measurement of leukotrienes, BALF (100 μL) was added to the 96-well plates, which were pre-coated with anti-LTD4 or -LTE4 antibody. The plates were sealed, incubated at 37 °C for 1.5 h, and washed three times. Biotinylated mouse LTD4 or LTE4 antibody (100 μL) was added, incubated at 37 °C for 1 h, and washed three times. Next, 100 μL of enzyme conjugate was added, incubated at 37 °C for 30 min, and washed five times. Color Reagent (100 μL) was added and developed at 37 °C. The reaction was quenched by the addition of 100 μL of Color Reagent C. 

For the measurement of histamine, 100 μL of BALF and 50 μL of histamine tracer were added to the 96-well plates. The plates were sealed, incubated at room temperature on the plate shaker for 1 h, and washed three times. Next, 200 μL of SA-HRP conjugate was added, incubated at room temperature on a plate shaker for 30 min, and washed three times. Next, TMB substrate solution (200 μL) was added and maintained for 30 min at room temperature on a plate shaker. The reaction was quenched by the addition of 50 μL of stop solution. 

The absorbance of Th2 cytokines, leukotrienes, and histamine on the plates was read at 450 nm with a microplate reader (Molecular Devices).

### 2.10. Culture of Asthmatic Bronchial Smooth Muscle Cells (ABSMCs)

Human asthmatic bronchial smooth muscle cells (HABSMCs) were purchased from Lonza (Walkersville, MD, USA). HABSMCs were cultured in Smooth Muscle Basal Medium-2 (SmBM-2) supplemented with SingleQuots kits containing growth factors (human epidermal growth factor, insulin, and human fibroblastic growth factor-B), gentamicin/amphotericin, and fetal bovine serum (FBS; Lonza). The cells were incubated at 37 °C with 5% CO_2_. The medium was replaced every 2 days. Mouse ABSMCs (MABSMCs) were isolated using the method developed by Wang et al. [26] with some modifications. Briefly, the tracheae of the asthmatic mice were excised, washed, and digested with 0.2% collagenase II (Worthington Biochemicals, Freehold, NJ, USA) and 0.05% papain (Worthington Biochemicals) for 30 min at 37 °C. The tissue was allowed to stand in a tube, and the supernatant was collected and centrifuged at 340× *g* for 5 min in a microcentrifuge (Eppendorf 5242R, Hamburg, Germany). The pellet containing MABSMCs was washed three times, resuspended in DMEM/Ham’s F12 (1:1) containing FBS to stop enzyme reaction, and then plated in 6-well plates. MABSMCs at passages 2–4 were used for the experiments.

### 2.11. Cell Viability Assay

Cell viability was determined colorimetrically using the 3-(4,5-dimethylthiazole-2-yl)-2,5-diphenyl tetrazolium bromide (MTT) reagent (Duchefa, Haarlem, Netherlands). The MTT assay procedures were performed as previously described [27]. Briefly, cells at the exponential phase were seeded (4 × 10^4^ cells/mL) in a 24-well plate. After 24 h of treatment with chemicals or SHH, 20 μL of 5 mg/mL MTT solution was added to each well (0.1 mg/mL) and incubated for 4 h. The supernatants were then aspirated, the formazan crystals in each well were dissolved in 200 μL of DMSO for 30 min at 37 °C, and the 24-well plates were read at 570 nm using a microplate reader (Molecular Devices). Data are expressed as the percentage of viable cells compared to the control.

### 2.12. Measurement of Intracellular Ca^2+^ Levels

Changes in the intracellular Ca^2+^ concentration ([Ca^2+^]_i_) were measured using the Ca^2+^-sensitive fluorescent indicator fluo 3-AM (Molecular probe, Eugene, OR, USA) and a confocal laser scanning microscope (IX70 Fluoview, Olympus), as previously described [28]. Briefly, MASMCs were incubated with 5 μM fluo 3-AM in a coverglass-bottom dish (SPL, Pocheon, Korea) for 45 min and washed three times with a serum-free medium. Fluorescent images were scanned every 5 s, at 488 nm with an excitation argon laser and at 530 nm with a long-pass emission filter. All scanned images were processed to analyze changes in [Ca^2+^]_i_ at the single-cell level. 

### 2.13. Cell Contraction Assay

A contraction assay of smooth muscle cells was performed using a cell contraction assay kit (Cell Biolabs, Inc., San Diego, CA, USA) as previously described [28]. Briefly, Collagen Gel Working Solution (CGWS) containing a collagen solution (bovine type I), 5X medium, and a neutralization solution was prepared and kept on ice. Mouse and human ABSMCs were resuspended in culture medium at 2 × 10^6^ cells/mL. A collagen lattice was prepared by mixing two parts of cell suspension and eight parts of cold CGWS. The cell–collagen mixture (0.5 mL) was placed per well in a 24-well plate and incubated for 1 h at 37 °C. After collagen polymerization, 1 mL of culture medium was added onto the collagen gel lattice. Cells in collagen gels were equilibrated for 2 days. The cells were treated with histamine, acetylcholine, and/or HSHH 1 h prior to releasing the stressed matrix. The 2,3-butanedione monoxime (BDM; 10 mM), a myosin inhibitor, was treated to the cells as a contraction inhibitor. The contraction was initiated by releasing collagen gels from the sides of the well in a 24-well plate. Images of the gels were scanned with an Epson Perfection 3170 photo scanner (Epson France, Levallois-Perret, France), and the changes in the collagen gel size (contraction index) were measured at the indicated times with a ruler, and quantified using ImageJ software (version 1.49, National Institute of Health, Bethesda, MD, USA). Collagen contraction was determined in at least quadruplicate under each condition. 

### 2.14. Statistics

Differences among groups were analyzed using a one-way ANOVA test with post hoc comparisons using Tukey’s test (SPSS18 software, SPSS Inc., Chicago, IL, USA). The significance was set at *p* < 0.05. Data are presented as the means ± SE.

## 3. Results

### 3.1. Alleviation of Ovalbumin (OVA)-Induced Airway Inflammation by High-Dose SHH (HSHH)

**Figure 2:**
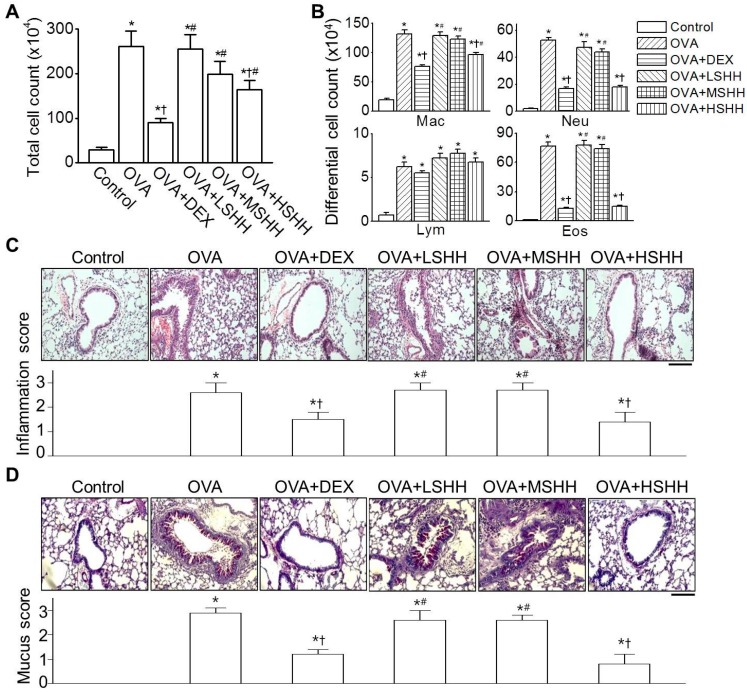
HSHH-induced reduction of inflammation in OVA-challenged asthmatic mice. (**A**) Total cell counts in bronchoalveolar lavage fluid (BALF); (**B**) Differential cell counts in BALF. Total cell counts were determined with 1 mL of BALF, and differential cell counts were assessed by Wright-Giemsa staining; (**C**) Representative images of Hematoxylin and Eosin (H&E) staining of lung tissue. The remarkable increase in cellular infiltration and airway thickness in the OVA group was not observed in the OVA + DEX nor OVA + HSHH groups. The scale bar represents 200 μm. The bar graphs show the inflammation score in each experimental group (see Materials and Methods 2.7); (**D**) Reduction of OVA-challenged goblet cell hyperplasia by HSHH. Representative images of periodic acid–Schiff (PAS) staining of goblet cells in the experimental groups. The scale bar represents 200 μm. The bar graphs show the number of PAS-positive mucus-producing cells (see Materials and Methods 2.7). In the bar graphs, data are shown as the means ± SE. * *p* < 0.05, compared to control. ^†^ *p* < 0.05, compared to OVA. ^#^ *p* < 0.05, compared to DEX.

BALF was collected from six experimental groups to analyze the inflammation levels. The OVA group showed a significant increase in the number of inflammatory cells in BALF (*p* < 0.05). In the OVA group, the number of macrophages, Neu’s, and Eos’s was high, compared to the control group (Figure 2A,B; *n* = 10). Administration of DEX significantly decreased the OVA-induced increase in the cell number of BALF cells (*p* < 0.05). Among mice that were administered with SHH, only HSHH significantly reduced the number of inflammatory cells increased by OVA (*p* < 0.05). Although DEX showed a higher inhibitory effect on the number of total inflammatory cells and macrophage compared to HSHH, the inhibitory effect of HSHH on the number of Neu’s and Eos’s in BALF was similar to that of DEX (Figure 2B; *p* > 0.05; OVA + DEX vs. OVA + HSHH). OVA-induced inflammation was analyzed in lung tissue. As shown in Figure 2C, lungs isolated from OVA-challenged mice showed a marked infiltration of inflammatory cells into the peribronchial area, compared to the control (*n* = 5). Administration of DEX or HSHH markedly reduced the inflammatory cell infiltration, but LSHH and MSHH showed insignificant changes in inflammatory cell infiltration, compared to OVA-challenged mice. Goblet cells that secreted mucus in each experimental group were observed using the PAS stain (Figure 2D; *n* = 5). The OVA group showed significant goblet cell hyperplasia (*p* < 0.05). Administration of DEX or HSHH reduced OVA-induced goblet cell hyperplasia, whereas LSHH and MSHH had no effect on OVA-induced goblet cell hyperplasia. 

### 3.2. Inhibition of OVA-Induced Increase in Total and OVA-Specific IgE in Serum by HSHH

**Figure 3:**
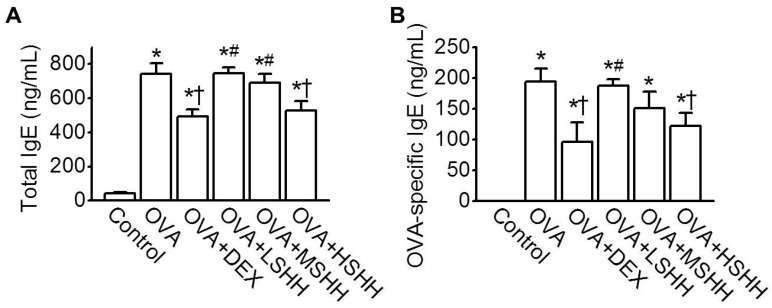
Inhibitory effect of HSHH on total and OVA-specific Immunoglobulin E (IgE) levels in serum. Levels of (**A**) total; and (**B**) OVA-specific IgE. Data are shown as means ± SE. * *p* < 0.05, compared to control. ^†^ *p* < 0.05, compared to OVA. ^#^ *p* < 0.05, compared to DEX.

The levels of total and OVA-specific IgE in serum were compared among experimental groups. As shown in Figure 3A, the level of total IgE significantly increased in the OVA group, compared to the control group (*p* < 0.05; *n* = 6). Administration of DEX or HSHH showed a significant reduction in the total IgE level compared to the OVA group (*p* < 0.05), while LSHH and MSHH did not affect the levels of total IgE increased by OVA. Changes in the OVA-specific IgE level in each experimental group were consistent with those of the total IgE levels (Figure 3B). The effects of HSHH on the levels of total and OVA-specific IgE were similar to those of DEX. 

### 3.3. Reduction of OVA-Induced Increase in Th2 Cytokines, Leukotrienes, and Histamine Concentrations in BALF by HSHH

**Figure 4:**
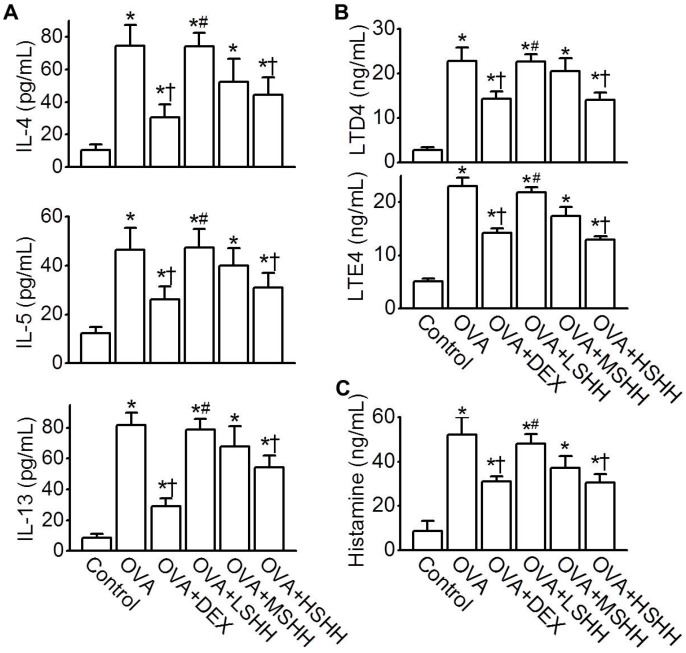
Inhibitory effect of HSHH on T helper cell type 2 (Th2) cytokines, leukotrienes, and histamine levels in BALF obtained from asthmatic mice. (**A**) Levels of IL-4, IL-5, and IL-13; (**B**) Levels of LTD4 and LTE4; (**C**) Levels of histamine. Data are shown as means ± SE. * *p* < 0.05, compared to control. ^†^ *p* < 0.05, compared to OVA. ^#^ *p* < 0.05, compared to DEX.

To identify the effect of SHH on the concentration of Th2 cytokines in BALF, the concentrations of IL-4, IL-5, and IL-13 were measured. As shown in Figure 4A, the levels of IL-4, IL-5, and IL-13 in BALF were significantly elevated in the OVA group, compared to the control group (*p* < 0.05; *n* = 10). Similarly to the administration of DEX, HSHH significantly reduced the OVA-induced increase in IL-4, IL-5, and IL-13 concentrations (*p* < 0.05). LSHH and MSHH did not affect the concentration of Th2 cytokines increased by OVA. The levels of leukotrienes (LTD4 and LTE4) and histamine in BALF, which are secreted from mast cells activated by Th2 cytokines, were measured (Figure 4B,C). OVA-induced increases in LTD4 and LTE4 in BALF were significantly reduced by the administration of HSHH (Figure 4B; *p* < 0.05; *n* = 10). OVA-induced histamine levels were also inhibited by HSHH, but not LSHH nor MSHH (Figure 4C; *n* = 10). The effect of HSHH on the OVA-induced increase in leukotrienes and histamine was similar to that of DEX.

### 3.4. Inhibition of Histamine-Induced Contraction in ABSMCs by SHH

**Figure 5:**
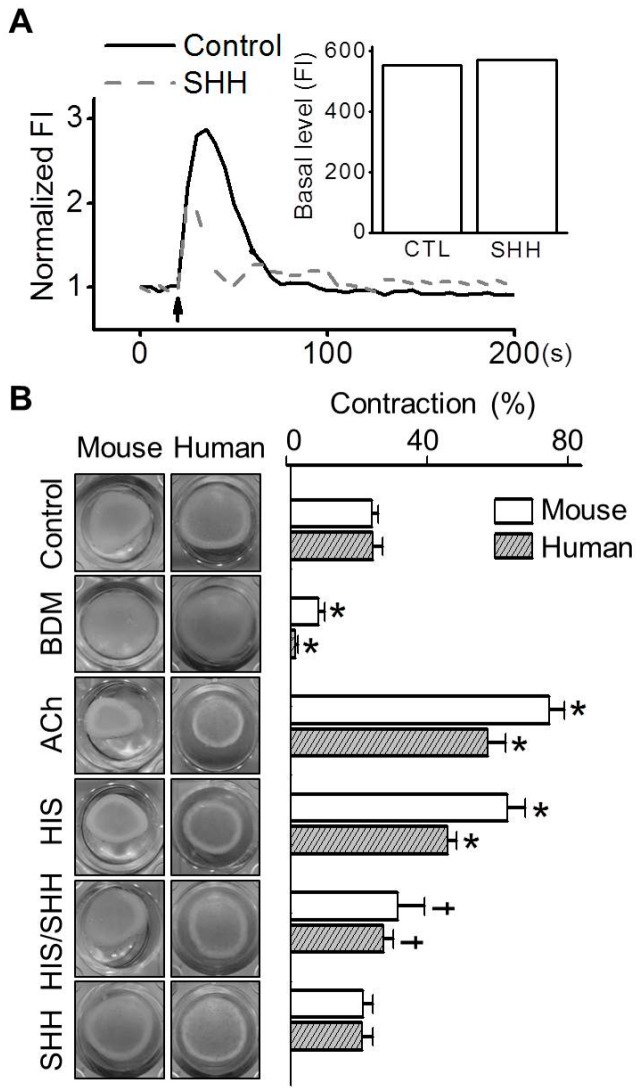
Reduction in histamine-induced contraction of ABSMCs by SHH. (**A**) Histamine-induced Ca^2+^ increase and reduction of the Ca^2+^ response by SHH in MABSMCs. Arrow indicates the addition of 100 μM histamine. Bar graph shows the effect of SHH on basal Ca^2+^ level in cells, which is the [Ca^2+^]_i_ before treatment with chemicals. FI represents the fluorescence intensity; (**B**) Representative images of collagen matrices in a collagen gel contraction assay. Cells were exposed to medium with or without chemicals and SHH for 24 h. Acetylcholine (ACh) and 2,3-butanedione monoxime (BDM) were used as a positive control and a negative control, respectively. Bar graphs show the contractility of ABSMCs induced by treatment with chemicals and SHH. Data are shown as means ± SE. * *p* < 0.05, compared to control. ^†^ *p* < 0.05, compared to histamine.

To investigate the effect of SHH on the contraction of smooth muscle cells, which are involved in airway narrowing and remodeling, Ca^2+^ changes and gel contractions were tested in ABSMCs. The concentrations of histamine and SHH without cytotoxicity in MABSMCs were determined using the MTT assay. No concentrations of histamine (1 to 100 μM) and SHH (10 to 100 μg/mL) tested affected the cell viability of MASMCs, and thus, 100 μM of histamine and 100 μg/mL of SHH were used for the additional experiments. Histamine, a potent constrictor of airway smooth muscles, induced an increase in the levels of [Ca^2+^]_i_, but pretreatment with SHH reduced the histamine-induced increase in Ca^2+^ levels in MABSMCs. SHH did not affect basal Ca^2+^ levels (Figure 5A; *n* = 10). The effect of SHH on histamine-induced contraction of ABSMCs was investigated. Addition of histamine to the gels containing MABSMCs and HABSMCs increased contractility by 2.6-fold and 1.9-fold, respectively, compared to the control (Figure 5B; *n* = 5). However, the combination of histamine and SHH significantly reduced histamine-induced contraction in MABSMCs and HABSMCs by 50.0 ± 4.6% and 39.6 ± 1.3%, respectively (*p* < 0.05). SHH alone did not affect the contractility of ABSMCs. Acetylcholine (ACh) and BDM were used as a positive control and a negative control for contraction, respectively.

### 3.5. No Changes in the Spleen and Thymus by HSHH

**Figure 6:**
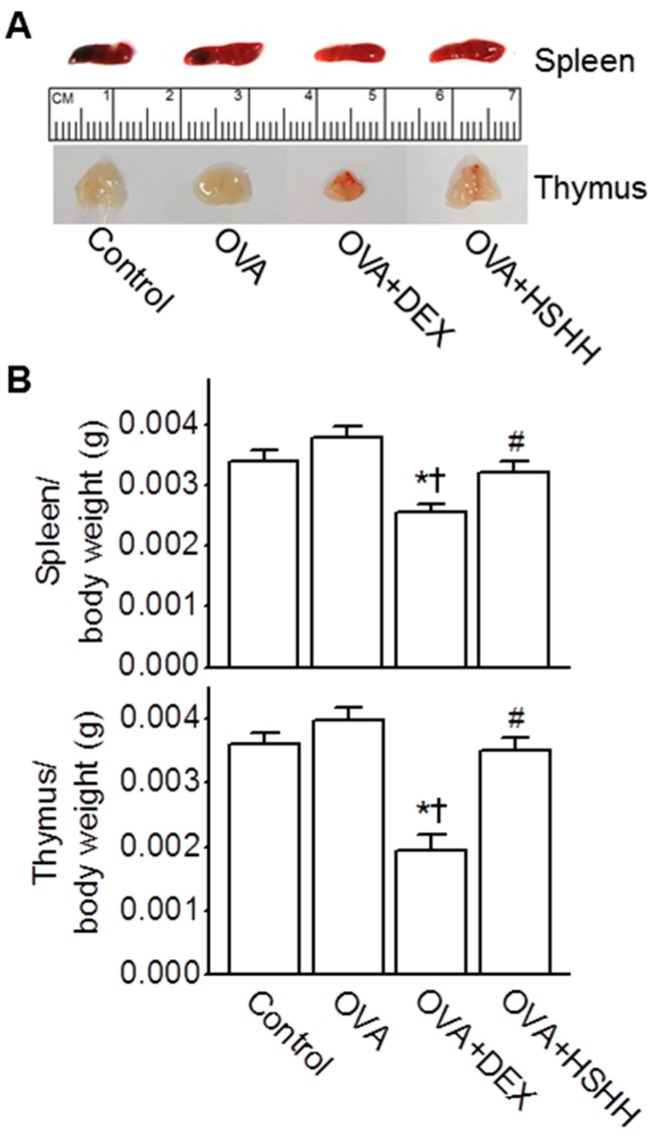
No changes in the spleen nor thymus weights by HSHH treatment. (**A**) Changes in the size of spleens and thymi in asthmatic mice; (**B**) Spleen and thymus weights in asthmatic mice. Bar graphs show the spleen and thymus weights isolated from the control, OVA, OVA + DEX, and OVA + HSHH groups. The ratios of organ weight to body weight were calculated. Data are shown as means ± SE. * *p* < 0.05, compared to control. ^†^ *p* < 0.05, compared to OVA. ^#^ *p* < 0.05, compared to OVA + DEX.

To determine whether HSHH showing an anti-asthmatic effect induced changes in immune organs, the spleen and thymus weights were evaluated in the experimental asthma model. The effect of HSHH was compared to that of DEX. As shown in Figure 6A, the sizes of the spleens and thymi were reduced in the OVA + DEX group, whereas no change was observed in the OVA + HSHH group, compared to the control and OVA group (*n* = 10). The ratios of thymus and spleen weights to body weights in the OVA + DEX group were also the smallest. However, the spleen and thymus weights were not affected by the HSHH treatment (Figure 6B; *n* = 10). 

## 4. Discussion

To the best of our knowledge, this study is the first report demonstrating that SHH reduces asthmatic parameters in an OVA-challenged allergic asthma model. The inhibitory effects of SHH on asthmatic parameters were evaluated in this study by reductions in the number of inflammatory cells; levels of total and OVA-specific IgE in serum; levels of Th2 cytokines, leukotrienes, and histamine in BALF; mucus production by goblet cells in lung tissue; and contraction of ABSMCs. Airway hyperresponsiveness, another asthmatic parameter, could not be evaluated in this study because of limitations of instruments to assess pulmonary function. Here, we indirectly present the contractility of ABSMCs as a parameter reflecting airway hyperresponsiveness. Among the three doses of SHH tested (low-dose: 0.1 g/kg/day; medium-dose: 0.5 g/kg/day; and high-dose: 1.0 g/kg/day), HSHH significantly reduced asthmatic parameters in the OVA-challenged allergic asthma model. MSHH slightly reduced asthmatic parameters with an insignificant difference from DEX treatment, but LSHH did not reduce asthmatic parameters in the OVA-challenged allergic asthma model. SHH showed a dose-dependent effect on the reduction of asthmatic parameters.

In the development of anti-asthmatic compounds, natural products showing antioxidant and anti-inflammatory activities have been considered as therapeutic agents [29,30,31] because both oxidative stress and inflammation are involved in the pathogenesis of asthma [32,33,34]. Bioactive molecules in SHH have demonstrated various bioactivities, such as antioxidant and anti-inflammatory activities, and the molecules have been purified from sea hare and identified as glycosaminoglycan [35], isoprenoids [36], monoterpenes [37], diterpenes [38], and alkaloids [39]. Even though the role of glycosaminoglycan (GAG), as a main component in SHH, has been unclear in the inflammatory process, several studies have demonstrated its critical role in inflammatory diseases [40,41,42]. In our previous study, however, GAG did not show anti-inflammatory effects on lipopolysaccharide (LPS)-activated RAW264.7 macrophage cells [7]. Carotenoids, lycopene, geraniol, and alkaloids have been studied for their anti-asthmatic effects on reducing oxidative damage [43], the infiltration of inflammatory immunocytes into the bronchoalveolar lavage [44], modulation of Th1/Th2 balance and activation of the nuclear factor erythroid 2-related factor 2 (Nrf2)/antioxidant response element pathway [45], and bronchodilation, respectively [46]. Diterpenes such as plaunotol and furanoditerpenes from *Croton stellatopilosus* leaves exhibit anti-inflammatory activity in LPS-activated RAW264.7 cells [47], and cafestol and kahweol, coffee-specific diterpenes, show significant free radical scavenging activity and lipid peroxidation inhibition [48]. SHH could be a promising resource due to its numerous potential components reducing asthmatic parameters, as described above, but the effect of some components on inflammatory disease is still controversial. The effect will likely be dependent on the concentration of molecules present in SHH. 

It has been reported that enzymic hydrolysate of marine organisms can enhance their bioactivities by releasing bioactive marine small peptides with various biological functions, such as antihypertensive, antioxidative, anti-inflammatory, antimicrobial, and immunomodulatory activities [49,50]. These bioactive peptides have more potency than their native protein in the treatment of diseases [51]. SHH used in this study was an enzymic hydrolysate, which is hydrolyzed by flavourzyme, a compound enzyme consisting of the incision enzyme and circumscribed enzyme. Treatment with flavourzyme effectively resects hydrophobic amino acids from the protein end [52]. The enzymic hydrolysate, SHH, could likely enhance their bioactivities and thus show a strong reduction of asthmatic parameters in OVA-challenged allergic asthma model. 

HSHH resulted in a reduction in Th2 cytokines. Suppressors of Th2 cytokines could not be overlooked in the development of anti-asthmatic compounds because excessive Th2 cytokines are a large risk factor for asthma [53,54]. The primary inflammatory lesion of asthma is accompanied by the accumulation of cluster of differentiation 4+ (CD4+) Th2 cells and Eos’s in the airway mucosa and its secretion of a series of cytokines, mostly IL-4, IL-13, IL-5, and IL-9. Th2 cytokines, particularly IL-4, induce a switch to IgE production by differentiating B cells [55]. The crucial function of IgE in allergic diseases, including asthma, has been proposed to be the sensitizing of mast cells by releasing several mediators, such as histamine, leukotrienes, cytokines, and chemokines [56]. In addition, Th2 cytokines also cause an increase in IgE receptor expression in mast cells [55,57]. Moreover, Th2 cytokines induce mucus metaplasia and mucin production [58]. The mRNA expression of mucin (MUC) 2 and MUC5AC mucin genes increases via direct stimulation of IL-4, IL-9, and IL-13 in airway epithelial cells, indicating that Th2 cytokines may act as crucial mediators in mucus production [58,59]. The histamine and cysteinyl leukotriene secreted from mast cells activated by Th2 cytokines increase [Ca^2+^]_i_, and thus the contractility of airway smooth muscle cells is increased [60,61]. Airway narrowing induced by airway smooth muscle contraction contributes to obstruction with a consequent reduced airflow, wheezing, and dyspnea in asthmatics [62]. HSHH reduced these asthmatic mediators and signals.

Immunosuppression is defined as an impairment of any components of the immune system, resulting in decreased immune function. Immunosuppression could be examined by checking the weight of immune organs and performing standard nonclinical histology and toxicology studies according to the “Guidance for Industry, Immunotoxicology Evaluation of Investigational New Drugs” (U.S. Department of Health and Human Services, 2002). Atrophy of the lymphoid organs, such as the thymus and spleen, has been examined for side effects on the immune system of drugs [63,64]. In the present study, the HSHH treatment group showed no changes in the thymus and spleen sizes and weights, compared to the control group. Thus, our results proved that SHH could reduce asthmatic parameters by its specific immune activity, but not by immunosuppression.

## 5. Conclusions

In conclusion, our results demonstrated that HSHH could successfully decrease airway inflammation, levels of total and OVA-specific IgE, mucus overproduction, and smooth muscle contraction, most likely via a reduction in Th2 cytokines and bronchoconstrictor mediators. The reduced levels of histamine, LTD4, and LTE4 inhibited the hypercontractile response in bronchial smooth muscle cells. Our findings suggest that *A. kurodai* hydrolysate could be used as a potential therapeutic agent and a functional food for asthma.
